# Nepal’s mental health system from public health perspective: a thematic synthesis based on health system building blocks

**DOI:** 10.1016/j.lansea.2025.100588

**Published:** 2025-04-30

**Authors:** Shishir Paudel, Anisha Chalise, Dhurba Khatri, Sujan Poudel, Aagya Khanal

**Affiliations:** a Kathmandu Institute of Child Health, Hepaliheight, Kathmandu, Nepal; b Center for Research on Environment, Health and Population Activities (CREHPA), Lalitpur, Nepal; c HERD International, Lalitpur, Nepal

**Keywords:** Mental health, Health system, Mental healthcare system, Mental health policy, WHO system framework, Six building block, Nepal

## Abstract

This study evaluates Nepal’s mental health system through the World Health Organization’s six building blocks: governance, financing, workforce, service delivery, essential medicines, and information systems. Despite policy advances toward integrating mental health services within primary care, significant challenges persist, including governance fragmentation, workforce shortages, insufficient funding, inconsistent availability of essential psychotropic medications, and a weak health information infrastructure. Federalization has introduced both opportunities and challenges for mental health governance across the different tiers of government. Stigma, resource constraints, and limited coordination further hinder service accessibility. This study identifies actionable priorities, including strengthening workforce capacity, expanding community-based services, enhancing inter-sectoral collaboration, and improving access to essential medicines. Addressing these issues through targeted policies and increased funding is crucial to build a more equitable and effective mental health system in Nepal, capable of addressing the rising burden of mental health disorders.

## Introduction

For nearly eight decades, the World Health Organization (WHO) defined health as “a state of complete physical, mental, and social well-being, and not merely the absence of disease or infirmity”.^1^ This holistic definition, introduced on 1948, underscores the importance of mental health as a crucial component. However, in many developing nations, including Nepal, mental health remains overshadowed by other health priorities.^2^, ^3^, ^4^ With limited health budgets, governments often struggle to meet the comprehensive health needs of their population and focus primarily on prevention and treatment of physical diseases and infections, neglecting the resources for mental health and well-being.^3^, ^4^, ^5^ As a result, a significant treatment gap persists for mental disorders globally, with four out of five people having mental health problems in lower-income countries deprived of effective treatment.^2^^,^^3^^,^^6^, ^7^, ^8^ For instance, a survey across 14 countries found 76.3%–85.4% of those with severe mental disorders in low-and middle-income countries (LMICs) do not receive any treatment.^6^

Mental health disorders contribute significantly to the global disease burden, with Disability-Adjusted Life Years (DALYs) increasing from 80.8 million in 1990 to 125.3 million in 2019.^9^ In 2017, depression was ranked as the leading contributor to global disability, accounting for 7.5% of all Years Lived with Disability (YLDs), while anxiety disorders ranked sixth, contributing 3.4% of YLDs.^10^ Furthermore, neuropsychiatric conditions contributes 13% of the total DALYs lost due to all diseases and injuries, which is projected to increase to 15% by 2030, with depression alone accounting for 5.7%.^11^

In Nepal, there has been growing concern over the mental health and well-being of its citizens in recent years. The country’s history of political instability, particularly a decade-long armed conflict that claimed over 16,000 lives, has left a lasting impact on the citizens’ mental health.^12^ During this period of civil unrest, the health system itself was severely impacted, hindering the delivery of health services, resulting in various adverse health outcomes.^13^ As Nepal undergoes political transformation, ongoing unrest, low socioeconomic status, social and cultural discrimination, and vulnerability to natural disasters such as earthquakes, floods, and landslides continue to heavily impact the mental health and well-being of Nepalese citizens.^14^, ^15^, ^16^

The National Mental Health Survey Nepal-2020 revealed nearly 10.0% of the adults experienced a mental disorder in their lifetime, whereas 4.3% illustrated signs of current mental disorder.^14^ Among adolescents, 5.25% had mental disorders, and 3.9% reporting suicidal thoughts.^14^ Likewise, the pilot study for this mental health survey reported the prevalence of mental disorders among the Nepalese population at 12.9%, where almost 10.0% of adults and 8.7% of children experienced suicidality.^17^ Although the National Mental Health Survey is the only large-scale study in Nepal, numerous community-based studies highlight high rates of anxiety, depression, and suicidal ideation among various groups, including civil war victims, disaster survivors, mothers, the elderly, working populations, and students.^15^^,^^18^, ^19^, ^20^, ^21^, ^22^ These findings revealed alarming mental health conditions in the Nepalese population.

Since the adoption of new constitution in 2015, Nepal has been transitioning to federalism, decentralizing power and introducing both opportunities and challenges in the health system. Federalization offers a crucial chance to reassess and rebuild the mental health system for equitable and effective service delivery across provinces and local levels. It also allows for addressing long-standing issues in governance, service delivery, and resource allocation, making a comprehensive evaluation essential. Therefore, this study aims to assess the current status of Nepal’s mental health system using WHO’s six building blocks to evaluate the structural and operational challenges within Nepal’s mental health sector. The WHO’s health system framework describes health systems in terms of service delivery, health workforce, information, medical products, vaccines and technologies, financing, and leadership/governance.^23^ Each building block represents a core function vital for an effective and equitable health system and provides a holistic approach to analyzing health systems for functionality, efficiency, and equity. This framework is particularly suitable for LMICs like Nepal, as it highlights systemic gaps and provides a basis for strategic planning and policy reform.^23^^,^^24^ Evidence was synthesized from various sources using the WHO’s six-building block framework. A total of 23 peer-reviewed journal articles, 18 national policy documents, 9 government reports, 17 pieces of grey literature, including non-governmental organization (NGO) reports, websites, and international reports and guidelines from organizations such as WHO and UN were reviewed. These sources were systematically analyzed to understand their contributions to the mental health system in Nepal.

**Search strategy and selection criteria.**

A comprehensive literature search was conducted using the PubMed, EMBASE, CINAHL, NepJol, and Google Scholar. The keywords such as “Mental Health Services” Or “Mental Health Plan” Or “Mental Health Policies” Or “Mental Health System” Or “Mental Health Act” Or “Mental Health Strategy” And “Nepal” were used. Additionally, government reports, acts, policies, and information from government official websites were reviewed to capture key national perspectives and documents that may not be available in academic databases. Experts working in health system of Nepal were consulted for unpublished reports and literature as well as to seek clarity on the unclear issues raised at the time of review. The literature search was initially conducted in January 2023, but as the study progressed, additional relevant literature and newly published documents were incorporated until March 2024 to ensure a comprehensive and up-to-date review and synthesis of Nepal’s mental health system. The detailed methodology is attached in the Appendix.

## Historical overview of mental health in Nepal

**Fig. 1:**
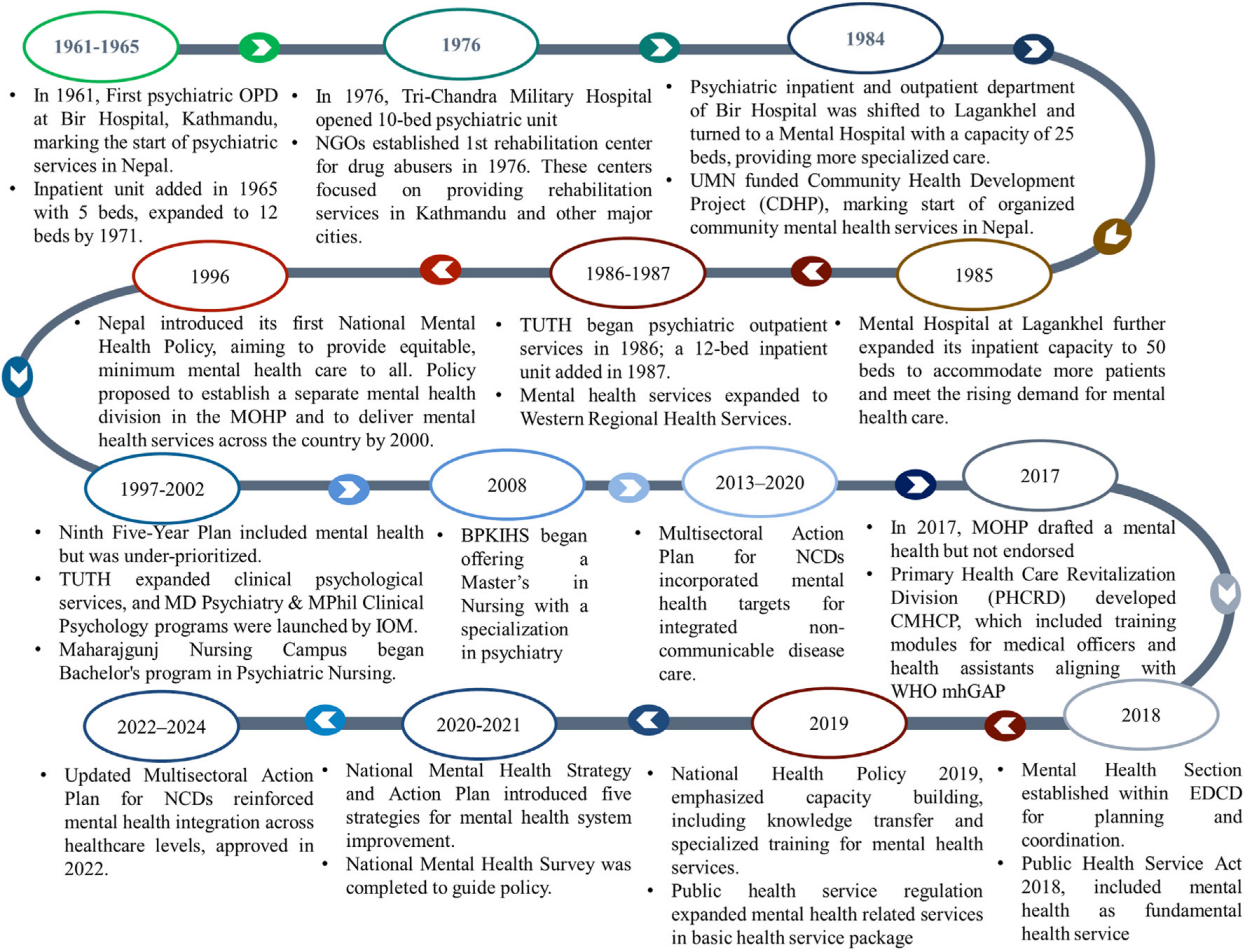
Mental health services in Nepal from inception to integration in different levels of care.

The progression of mental health services and policy development in Nepal has been marked by several key milestones, ranging from the establishment of the first psychiatric outpatient service in 1961 to recent policy initiatives integrating mental health into broader healthcare frameworks. These developments are illustrated in Fig. 1, which provides a chronological overview of major advancements in Nepal’s mental health system, including the expansion of psychiatric care facilities, the introduction of mental health policies, and efforts to integrate mental health services into national health programs.^25^, ^26^, ^27^, ^28^, ^29^, ^30^, ^31^, ^32^, ^33^, ^34^, ^35^, ^36^, ^37^, ^38^, ^39^

## Mental health services in Nepal: six building block**s**

### Leadership/governance for mental health

The NCD and Mental Health Section under the EDCD of the Department of Health Services (DoHS), Ministry of Health and Population (MoHP) is a governing body responsible for the overall planning, coordination, and implementation of national mental health-related programs and services.^36^ Additionally, the Curative Service Division, DoHS oversees the provision of all health-related services, including mental health services nationwide.^40^ The National Health Training Centre (NHTC) coordinates all health training activities, including those related to mental health.^41^ The National Health Education, Information and Communication Centre develops and disseminates information, education, and communication (IEC) materials for health education, including mental health-related IEC materials.^42^ These agencies collaborate with government bodies and NGOs to provide mental health services in Nepal, adhering to relevant policies and regulations related to mental health in Nepal.

In 2019, a new National Health Policy (NHP) was endorsed, which automatically nullified all previous health policies, and set forth the need for new mental health policy.^37^ The NHP 2019, specifically section 6.17.5, proposed a strategy to integrate mental health services into the broader health system and emphasized access to mental health services through primary hospitals, including specialized training for mental health professionals.^37^ There is no standalone mental health policy or national mental health act in Nepal, but mental health components are reflected in various other acts and guidelines, indicating an integrated but fragmented approach to mental health governance. The Public Health Services Act, 2018, section 3, includes mental health care as a fundamental health service.^43^ Additionally, the Public Health Service Regulations broadened the range of mental health services that are part of the Basic Health Service and Emergency Health Service Packages,^44^^,^^45^ showcasing efforts to ensure mental health services are part of essential health coverage. This initiative was followed by the National Mental Health Strategy and Action Plan in 2020, which outlined five strategies for improving mental health systems.^46^^,^^47^ This action plan emphasize integration, capacity building, and service user engagement.^46^ The strategic action plan outlines provisions for free primary care mental health services across the country, integrating mental health services into primary healthcare, enhancing mental health services at the secondary healthcare level, and ensuring the involvement of service users in policy development and planning.^46^ This governance approach aligns with WHO principles by fostering inclusivity and participatory decision-making. The updated Multisectoral Action Plan for NCDs (2021–2025), also reinforced mental health services within all healthcare levels.^30^

Despite these advancements, there is a lack of clear accountability framework and leadership within the mental health sector, which hampers the effective implementation of policies and plans. Furthermore, coordination between federal, provincial, and local government levels, as well as across different sectors such as health, education, and social services, remains weak, affecting the integrated delivery of mental health services. While the vision of integrating mental healthcare into the primary healthcare system is promising, there remain substantial gaps in governance mechanisms and human resources at both the provincial and local levels.^48^ Addressing these requires political commitment, dedicated resources, and a sustainable governance framework in line with WHO guidelines.

Although there is no updated mental health policy after 1996, mental health care in Nepal is governed by different policy documents after federalism. This has further expanded after the adoption of federalism which has created both opportunities and challenges to reassess and reconstruct the mental health system across the three tiers of governments. The constitutional mandate aims to decentralize power and enhance governance by bringing it closer to the people, allowing provincial and local governments to formulate their policies and strategies within their respective jurisdictions while adhering to the constitution and national policies, acts, and strategies. In this regard, each province has developed its unique approach to mental health care, responding to local priorities and challenges (Table 1).

**Table 1:** Policies from Provincial governments to address mental health care.

Province	Policy	Description
Koshi Province	Public Health Policy 2020^49^	Section 2, guarantees free access to mental health services as a fundamental right for all citizens.
Madhesh Province	Health Sector GESI Strategy 2022^50^	Section 5, specifies basic health services will be provided to individuals with physical disabilities, encompassing various initiatives related to mental health. These include updating fact sheets on mental health, integrating mental health into a “One Health” approach, and emphasizing needs assessments, counseling skills, and feedback mechanisms to enhance mental health support.
Bagmati Province	Provincial Health Policy 2023^51^	Policy 5.7, outlines plans to expand specialized mental health services in provincial hospitals and establish multi-sectoral initiatives for suicide prevention.
Gandaki Province	Provincial Health Policy 2022^52^	Prioritizes prevention, control, and treatment of mental health conditions alongside other NCDs and diseases linked to climate change.
Lumbini Province	Provincial Health Policy 2021^53^	Section 4, advocates for the expansion of both modern and Ayurvedic practices for the prevention and management of mental health issues, along with psychosocial counseling and treatment services
Karnali Province	Health Sector Strategic Action Plan (2024/25–2031/32)^54^	Addresses the needs of homeless individuals with mental health challenges, promoting development of rehabilitation centers through collaborative partnerships across sectors
Sudurpashchim Province	Provincial Health Policy 2022^55^	Emphasizes establishment of mental health divisions within provincial hospitals and the provision of specialized services at the district level.

This decentralized approach illustrates a commitment to strengthening mental health governance in Nepal, with each province developing strategies that address local needs while upholding the goals of the national health framework.

### Mental health workforce

A well-trained and adequately supported workforce is crucial for effective health service delivery. Although there is no universally accepted ideal ratio of mental health professionals to population, like the established ratio for doctor-to-patient ratio (1:1000),^24^ the WHO indicator suggests the number of psychiatrists or human resources working in the mental health sector can be assessed per 100,000 population.^23^ Applying this metric, Nepal faces a critical shortage of mental health professionals, including psychiatrists, psychologists, psychiatric nurses, and social workers, creating barrier for provision of services and access to care. The WHO estimates that Nepal has around 147 psychiatrists, with only three specializing in child psychiatry,^34^ which is now reported to be five. However, the study from Rai et al. (2021) offered slightly higher estimates, reporting about 200 psychiatrists, 30 clinical psychologists, 50 psychiatric nurses, 700 trained psychosocial counselors, and more than 300 community-based psychosocial workers in Nepal in 2020.^56^ This status suggests that Nepal has 0.68 psychiatrists and 0.17 psychiatric nurses per 100,000 people.^56^ Even with this higher estimate, the number remains insufficient for Nepal. Compounding these challenges, a substantial portion of mental health professionals i.e., around 110 psychiatrists, are engaged in private practice, while approximately 700 lay counselors are operating within the public sector.^56^

Nepal’s training capacity for mental health professionals is limited. While psychiatry programs produce 15–20 new psychiatrists annually, clinical psychology training is confined to a single institution, graduating only 2–3 professionals per year.^34^ Moreover, there are no training programs for mental health sub-specialties, such as addiction, child, or geriatric psychiatry, further restricting the diversity and depth of the mental health workforce.^56^ To address these gaps, ongoing professional development, mentorship programs, and career support are essential for retention and workforce growth.

The NHTC has translated and adapted the mhGAP Intervention Guide (mhGAP-IG), version 2.0, to create a comprehensive training program for mental health professionals in Nepal. This program consists of six modules, each tailored to a specific group of healthcare workers: Module 1 trains nurses in psychoeducation and behavioral activation for depression, with an optional module on alcohol-related counseling. Module 2 has two sub-modules for medical doctors and paramedics, focusing on managing mental, neurological, and substance use (MNS) disorders in primary care. Module 3 trains medical doctors, nurses, and paramedics in managing developmental, behavioral, and emotional disorders in children and adolescents. Module 4 prepares Female Community Health Volunteers (FCHVs) for MNS disorder screening and referrals, strengthening community-based mental health support. Module 5 trains public health professionals in designing and evaluating community mental health programs. Module 6 provides para-professional counselor training for health assistants and nurses in government facilities.^47^^,^^57^^,^^58^

Additionally, various organizations contribute to capacity building through mental health courses and counseling training.^47^^,^^59^^,^^60^ In 2022, an estimated 1700 health workers, including 215 program managers, 584 primary care providers, and 938 FCHVs, were trained in mental healthcare.^59^ This initiative aims to enhance the skills and knowledge of existing healthcare workers, thereby improving the overall delivery of mental health services in Nepal. However, frequent staff transfers and inadequate mental health training for general health workers hinder service continuity.^48^ Considering the rising mental health burden in Nepal and limited human resources to address this crisis, there is a clear need to emphasize human resource planning and management to develop comprehensive strategies specifically tailored to address the mental health workforce needs in Nepal. Currently, limited formal policies are focusing on strategic human resource management within the mental health sector. Developing such policies would align with the WHO’s framework for sustainable health systems, ensuring mental health workforce is adequately planned, managed, and supported to meet the population’s needs.

### Service delivery

Mental health service delivery in Nepal faces significant challenges, including limited infrastructure and accessibility. The country has only one public-sector psychiatry hospital (50 beds) and four private-sector psychiatry hospitals.^34^^,^^61^ Mental health services are primarily offered by psychiatry units at 19 medical colleges, 27 provincial and government hospitals, and 364 private hospitals, yet coverage remains inadequate.^34^ According to the Health Facility Survey 2021, only 25.2% of health facilities (394 out of 1565) offer mental health treatment services, with just 27.0% having proper guidelines, only 16.0% having staff with recent training in mental health care, and fewer than half maintaining stocks of essential medications.^61^ It is estimated that approximately 500 beds are available for mental disorders throughout the country, equating to 1.5 beds per 100,000 people.^56^ To address the service gap, the MoHP developed and implemented the Community Mental Health Care Package 2017, which integrates mental health services into primary care in alignment with the mhGAP.^34^^,^^56^ This model aims to manage common mental health disorders, including depression, anxiety, alcohol use disorder, and child and adolescent mental and behavioral disorders at the primary care level.^34^ Furthermore, the MoHP has made a mandate that general hospitals with over 200 beds should establish functional mental health units.^58^ In 2022, the “*Khulla Mann*” District Mental Health Care Program was introduced to build upon and replace the 2017 Package.^58^

The “*Khulla Mann*” program aims to enhance primary mental health services through a people-centered, needs-based approach by integrating mental health care into district hospitals, Primary Health Care Centers (PHCCs), and health posts, while linking them with community resources to promote mental health equity.^58^ However, Nepal faces a severe shortage of psychiatrists, making nationwide deployment unfeasible and potentially compromising service quality. A more practical interim strategy in the current context could be as suggested by Upadhaya and colleagues (2017), which involves a task-sharing approach where trained primary healthcare workers manage mental health medications and provide counseling services, as recommended by mhGAP.^48^ This can be further supported by telemedicine and mobile health (mHealth) interventions to reach underserved populations. However, this should be a temporary measure until Nepal produces sufficient numbers of specialized mental health professionals, such as psychiatrists and psychologists.

Nepal is one of seven countries implementing the WHO Special Initiative for Mental Health (WHO SIMH), which seeks to ensure universal health coverage in mental health services.^58^ Despite ongoing efforts, mental health services remain poorly integrated into other public health programs such as HIV/AIDS, tuberculosis, and maternal and child health.^34^ Additionally, although mental health components was included in the Multisectoral Action Plan for the Prevention and Control of NCDs 2013–2020,^30^ its implementation has been limited.^34^^,^^59^ The NGOs and external developmental partners have played a crucial role in bridging service gaps and extending mental health services beyond formal healthcare system, particularly in underserved and crisis-affected communities, by supporting individuals impacted by civil conflicts, the COVID pandemic, earthquakes, and other disasters.^34^^,^^35^^,^^56^^,^^58^^,^^62^ The MoHP is continuously collaborating with NGOs to expand community mental health programs, contributing to the integration of mental health services into primary healthcare.^35^^,^^47^^,^^58^ Some NGOs are closely collaborating with the government to strengthen mental health services through capacity building, training healthcare professionals, developing community-based mental health programs, and rehabilitating individuals with mental illness. They also engage in awareness campaigns and policy advocacy to improve access, particularly for vulnerable populations.^27^^,^^62^ Many medical colleges and academic institutions are also involved in mental health programs, integrating mental healthcare into district hospitals as part of medical and psychiatry residency programs.^63^ Furthermore, recognizing the need for stronger public-private partnerships (PPP), the government has begun co-funding programs with NGOs to provide mental health services for homeless individuals with mental illnesses.^63^ This structured collaboration between the government, NGOs, and academic institutions is essential for expanding mental health services and ensuring equitable access across Nepal.

Under the Basic Health Service Package (BHSP) 2018, treatment for depression, psychosis, alcohol use disorder, and epilepsy is provided free of cost.^45^ The National Mental Health Care Program (2022) further supports primary care-oriented mental health services, providing guidance for program implementation across all government levels.^58^ Despite these efforts, barriers such as poor accessibility, poverty, and stigma continue to hinder service utilization.^48^^,^^56^ Mental illness remains highly stigmatized in Nepal, with individuals seeking care often labeled as “crazy” or “mad”, which deters many from accessing the help they need.^7^^,^^14^ The mental health survey reported that 10.5% of adults with mental disorders feared being labeled ‘crazy’ if they sought care, while 11.7% felt embarrassed or ashamed to some extent.^14^ These issues highlight the urgent need to address mental health stigma and improve service delivery. Addressing these challenges requires expanding service delivery points, enhancing the quality of care, and integrating mental health services more effectively within the general health system, ensuring they are accessible, affordable, and acceptable to all segments of the population, particularly the most vulnerable.^34^

### Essential medicine and technologies

Access to essential psychotropic medications and appropriate mental health technologies is a critical component of Nepal’s mental health service delivery framework. The timely availability of medications is essential for managing various mental health conditions. A range of psychotropic medications, including antipsychotics, antidepressants, anxiolytics, mood stabilizers, and antiepileptics, are technically available at health facilities at all levels across Nepal.^34^ The BHSP and the National list of essential medicines include key medications for mental and behavioral disorders, such as diazepam, fluphenazine, amitriptyline, chlorpromazine, carbamazepine, sodium valproate, and risperidone.^45^^,^^64^ However, their availability seems to be limited on paper, and there seems to be an inconsistent supply and unavailability of the listed medications for mental and behavior disorders at the operational level.^48^^,^^58^ This inadequate and irregular supply of psychotropic medications at the local level is one of the significant barriers to effective mental health service delivery. Patients frequently pay out-of-pocket for medications due to supply chain disruptions, which disproportionately affect those in rural and underserved areas.^48^^,^^58^ Logistical challenges such as transportation issues, inadequate storage facilities, and inefficient supply chains further contribute to the problem, hindering continuous access to essential medications.

In Nepal, psychotropic medications can be prescribed by registered medical doctors, which typically include psychiatrists and general practitioners. However, in the absence of such specialists, the health assistants working in primary health care settings are authorized to prescribe certain medications, provided they have received specific training and adhere to established government protocols.^34^ While this approach helps bridge the mental health treatment gap, it requires robust training programs and ongoing supervision to ensure safe and effective prescribing practices.

In terms of mental health technologies, Nepal has limited resources, with only a few initiatives supporting telepsychiatry, electronic health records, and digital mental health tools. One notable initiative is the tele-mental health outpatient department at the Child and Adolescent Psychiatry Unit of Kanti Children’s Hospital and the Adolescent Mental Health Unit (AMHU) at the Mental Hospital, which provide tele-mental health services to expand access for adolescents in remote and underserved regions.^65^^,^^66^ Despite these advancements, telemedicine for mental health services remains underdeveloped and is not yet fully integrated into Nepal’s healthcare system. Expanding these technologies could significantly enhance mental health service accessibility and quality, particularly for populations in hard-to-reach areas. Improving access to essential psychotropic medications and mental health technologies in Nepal requires increased budget allocation, strengthened supply chain management, expanded training for non-specialist health workers, and better integration of digital health tools for remote care, ensuring equitable mental health services for all.

### Health financing

Health financing for mental health sector in Nepal remains a challenge, reflecting systemic issues within healthcare system. The health sector’s contribution to the national Gross Domestic Product (GDP) is relatively low, accounting for only 1.8% in 2021/2022.^67^ This lower contribution appears to correlate with a lack of political support and commitment to health, despite compelling arguments that health sector should be viewed as a crucial investment rather than expenditure. While the national budget for health grew modestly from NPR 40.6 billion in Fiscal year (FY) 2016/17 to NPR 69.38 billion in FY 2022/23,^68^ it remains insufficient. In FY 2023/24, only 4.95% of the national budget was allocated to SDG 3,^69^^,^^70^ while the overall health sector share for the same period is 5.8%.^67^ This is significantly below the target set by the National Health Sector Strategy, which aims to allocate 10% of the national budget to health.^71^

These financial constraints are particularly evident in the mental health sector. The actual proportion of the health budget allocated to mental health sector is not publicly stated,^48^ and due to lack of this transparency there are issues that hinder funding assessments. Available data shows a decline in budget allocation, with only 0.2% of the total health budget dedicated to mental health sector in 2020, compared to 0.8% in 2008.^56^ This reduction is concerning given the rising demand for mental health services. Although the BHSP includes mental health services at various levels of care,^45^ inadequate financing hampers implementation. Limited budget allocation and low prioritization contribute to irregular psychotropic medication supply at the district level, leading to frequent stock-outs.^48^ This underfunding hampers the sustainability of mental health programs at the district and local levels.

A qualitative study found that policymakers and planners perceive the mental health sector budget as largely supporting the national mental hospital, with minimal funding for community-based programs.^48^ This is concerning as it limits the availability of mental health services beyond hospital settings. It has been estimated that individuals with mental disorders spent NPR 16,053 on average annually for its management, with additional transportation and related costs of NPR 4146 and NPR 3,460, respectively, posing a significant financial burden given Nepal’s low per capita income. The high out-of-pocket costs, coupled with the lack of comprehensive financial protection, create substantial economic strain on affected individuals and households.^14^ To improve mental health financing, Nepal must enhance budget transparency, align funding with service demand, prioritize community-based programs, and revise social health insurance to expand mental health service coverage and reduce out-of-pocket costs.

### Health information system

Integrating mental health into Nepal’s national health information system presents significant challenges, primarily due to the low case detection rate and the limited knowledge and MHL among health workers. The annual report suggests that mental health service data has historically been underprioritized in the Health Management Information System (HMIS), lacking indicators and tracking systems. Although recent efforts introduced a new patient register for NCDs and mental disorders to capture essential data, challenges persist in data quality, completeness, and facility enrollment.^58^ Mental health information in Nepal is reported in two different formats, depending on the type of health facility. In hospitals and PHCCs, mental health-related data is recorded based on diagnoses made by medical doctors, allowing for comprehensive documentation and reporting of all potential diagnoses within these facilities. However, at the Basic Health Centers (BHCs), health workers must select from a set of 15 pre-coded diagnosis options that cover most priority MNS conditions.^34^ In BHCs, the critical issue is that many health workers lack the necessary knowledge and skills to accurately diagnose and report from the provided list. This skill gap leads to significant underreporting of mental health conditions.

In 2022, WHO Nepal and the Government of Nepal collaborated to integrate eight mental health indicators into the HMIS,^59^ to improve reporting of mental health related data across health system and facilitate policy planning. However, many policymakers and district-level planners are unaware of the types of mental health information available through the HMIS.^48^ This lack of awareness limits the effective use of available data, underscoring the need for improved dissemination and utilization of such information. Recent initiatives to integrate mental health services at the primary care level have prioritized different clinical conditions, some of which are not included in the diagnostic checklist provided to BHCs.^34^ This mismatch has further aggravated the issue of underreporting and has resulted in incomplete mental health data. Additionally, the quality of data from hospitals often does not meet desired standards, as the information is not routinely calculated or periodically reviewed for accuracy and completeness. Suicide-related information poses an even greater challenge, being dispersed across various sources such as vital statistics, hospital records, and police reports, making comprehensive data collection and analysis difficult.^34^ To strengthen Nepal’s mental health information system, it is crucial to enhance health workers’ capacity in diagnosis and reporting, standardize data collection, and conduct regular data reviews to ensure accuracy and completeness. Addressing the observed challenges is crucial for building a more robust health information system that can better support the planning, implementation, and evaluation of mental health services across the country.

## Implications for policy and practice

This study highlights key gaps in Nepal’s mental health system across WHO’s six building blocks, emphasizing their interconnections. Developing a more equitable system requires strong leadership to align national, provincial, and local policies, with accountability and multi-sectoral collaboration across health, education, and social service sectors. Expanding access to care through workforce training, task-sharing, and community-based services is essential, as is increasing the budget in mental health sector to reduce out-of-pocket costs and ensure medication availability. Strengthening primary care integration, task-sharing initiatives, and digital health technologies, including telemedicine, can improve service accessibility, especially in rural areas to close treatment gaps. Such initiatives could significantly improve service accessibility and quality, especially in the absence of specialized professionals. Furthermore, enhanced health information systems and mental health research are crucial for informed decision-making. Additionally, reducing stigma through community awareness campaigns could increase service uptake and improve mental health outcomes.

Despite ongoing efforts to strengthen human resources and integrate mental health services into primary care, sustainability and nationwide coverage remain challenging. There is a need to further institutionalize these initiatives by addressing workforce shortages, improving capacity-building mechanisms, and ensuring their integration at all levels of care. Additionally, a dedicated Mental Health Section within the health system, which would focus exclusively on mental health operations, policy execution, and program monitoring, would further help in strengthening the mental health service advocacy and support system. Establishing such a section would not only streamline mental health governance but also necessitate the recruitment of mental health-specific workforce to support strategic planning, coordination, and service delivery improvements.

Despite being one of the few studies to provide a structured analysis of Nepal’s mental health system through the WHO building blocks, this study is not without certain limitations. While it offers a comprehensive overview incorporating recent acts, policies, and updates, some recent or rapidly changing policy developments across different government tiers may still be missed due to Nepal’s evolving federal system. Additionally, though professionals working within the health systems were consulted during review process for their insights and to clarify certain issues, the study relied heavily on secondary data sources which might have missed the direct perspectives of different stakeholders engaged in the mental health sector. Finally, although the WHO building blocks framework gives a clear and structured approach, it may limit a detailed exploration of Nepal’s unique social, cultural, and economic factors that also shape mental health service delivery. These social, cultural, and economic factors that could affect mental health service delivery could be further explored by future exploratory studies based on primary data.

## Conclusion

The WHO building block framework helped analyze the current mental health system of Nepal and identify key areas for improvement. Governance, workforce shortages, underfunding, and limited information systems have created gaps that prevent many from accessing mental health services. By taking a coordinated approach that addresses each part of the health system, Nepal can build a more accessible and resilient mental health system. Long-term efforts that prioritize training, financing, service delivery, and essential resources can help make mental health services an integral part of Nepal’s healthcare landscape and ensure better mental health outcomes for all.

## Contributors

Shishir Paudel and Anisha Chalise: conceptualization, project administration, Writing—original draft, writing–review & editing. SHP and AC contributed equally. Dhurba Khatri, Sujan Poudel: project administration, writing–review & editing. DK and SUP contributed equally. Aagya Khanal: writing–review & editing. All authors critically reviewed and agreed on the final version of the manuscript.

## Declaration of interests

We declare no competing interests.

## Appendix A Supplementary data

Appendix

Summary in Nepali Language
