# Supplementary material for: Nepal’s mental health system from public health perspective: a thematic synthesis based on health system building blocks
Source: Lancet Reg Health Southeast Asia. 2025 Apr 30;36:100588. doi: 10.1016/j.lansea.2025.100588 (PMC12076720; doi:10.1016/j.lansea.2025.100588)
Supplement: Appendix [file mmc1.docx]

**Appendix: Detailed methodology**

**Research Question**

What are the key gaps and challenges in the delivery of mental health services in Nepal based on WHO six building blocks framework?

**Data Screening and Selection Process**

A rigorous screening process was employed to ensure the inclusion of relevant literature. A total of 112 articles were retrieved from the database and managed in EndNote X7 reference management software. After removing duplicates, 82 records were screened for relevance based on titles and abstracts. Of these, 46 full-text articles were assessed for eligibility based on inclusion and exclusion criteria. Literature providing insights into Nepal’s mental health system, specifically illustrating any of the WHO’s six health system building blocks, was included. This encompassed sources such as national surveys, policy documents, relevant review papers, and national reports. Studies focused solely on mental health issues, such as prevalence rates or clinical aspects unrelated to health system components, were excluded. Literature published in both English and Nepali was included, with no date restrictions, to ensure a comprehensive scope of relevant data. Additionally, government policies, acts and regulations were explored through other sources. Ultimately, 67 documents were reviewed in detail, forming the basis for the thematic synthesis.

**Data Synthesis**

Collected data were organized into themes corresponding to each of the WHO building blocks. For each building block, information was extracted, analyzed, and organized to highlight both systemic challenges and areas where progress has been made. Insights from national and provincial health policies were integrated to contextualize the findings.

**Expert Consultation**

To supplement the literature, former and current government personnel from key divisions, including the Policy, Planning and Monitoring Division (PPMD) of the Ministry of Health and Population (MoHP), the Epidemiology and Disease Control Division (EDCD) of the Department of Health Services (DoHS), the Curative Service Division (CSD) of DoHS, and the National Health Training Centre (NHTC) were consulted for further insight into the context. In addition, experts actively working in Nepal’s mental health sector provided insights into policy implementation, challenges, and system gaps.
