# Supplementary material for: Nepal’s mental health system from public health perspective: a thematic synthesis based on health system building blocks
Source: Lancet Reg Health Southeast Asia. 2025 Apr 30;36:100588. doi: 10.1016/j.lansea.2025.100588 (PMC12076720; doi:10.1016/j.lansea.2025.100588)
Supplement: Summary in Nepali Language [file mmc2.pdf]

सार

यो विषयगत समीक्षाले नेपालको मानसिक स्वास्थ्य प्रणालीलाई विश्व स्वास्थ्य संगठनका छ वटा आधार स्तम्भहरुः नेतृत्व तथा सुशासन, वित्तीय व्यवस्थापन, मानव संसाधन, सेवा प्रवाह, आपूर्ति व्यवस्थापन, र स्वास्थ्य सूचना प्रणालीको आधारमा मूल्यांकन गरेको छ। प्राथमिक स्वास्थ्य सेवामा मानसिक स्वास्थ्यलाई एकीकृत गर्ने दिशामा नीतिगत प्रगति भएतापनि, नेतृत्वको खण्डीकरण, स्वास्थ्य जनशक्तिको अभाव, अपर्याप्त आर्थिक स्रोत, आवश्यक मनोचिकित्सा औषधिहरूको अनियमित उपलब्धता, र कमजोर स्वास्थ्य सूचना पूर्वाधार जस्ता महत्त्वपूर्ण चुनौतीहरू यथावत नै छन्। संघीय संरचनाले विभिन्न तहका सरकारहरूकालागि मानसिक स्वास्थ्य सेवा व्यवस्थापन गर्ने अवसर र चुनौती दुवै प्रदान गरेको छ। यद्यपि समाजमा रहेको मानसिक समस्या प्रतिको नकरात्मक दृष्टिकोण, स्रोत साधनको कमी, र सरोकारवाला निकाय बिचको समन्वयको अभावले सेवाको पहुँचमा थप बाधा पुर्याएको छ। जनशक्तिको क्षमता अभिवृद्धि, समुदाय-आधारित सेवाहरूको विस्तार, बहु-क्षेत्रीय समन्वयको प्रवर्द्धन, र आवश्यक औषधिहरूको सहज पहुँच यस समीक्षाले पहिचान गरेका केही कार्यान्वयन योग्य प्राथमिकताहरू हुन्। नेपालमा बढ्दै गएको मानसिक स्वास्थ्य समस्याको भार बहन गर्न सक्षम, मानसिक स्वास्थ्य लक्षित नीति र पर्याप्त वित्तीय लगानी मार्फत उक्त चुनौतीहरु सम्बोधन गर्नुका साथै समान र प्रभावकारी मानसिक स्वास्थ्य प्रणाली निर्माण गर्न अत्यावश्यक रहेको छ।
